# Supplementary figures and images for: The use of social robots with children and young people on the autism spectrum: A systematic review and meta-analysis
Source: PLoS One. 2022 Jun 22;17(6):e0269800. doi: 10.1371/journal.pone.0269800 (PMC9216612; doi:10.1371/journal.pone.0269800)

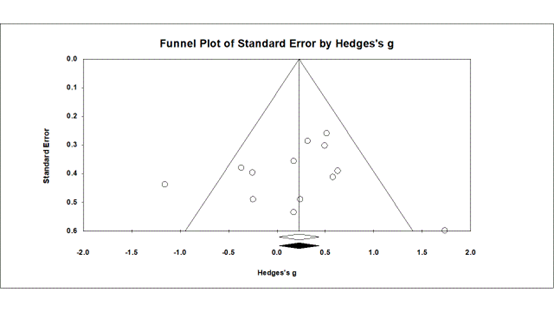

Supplement: S1 Fig — (TIF) [file pone.0269800.s001.tif]

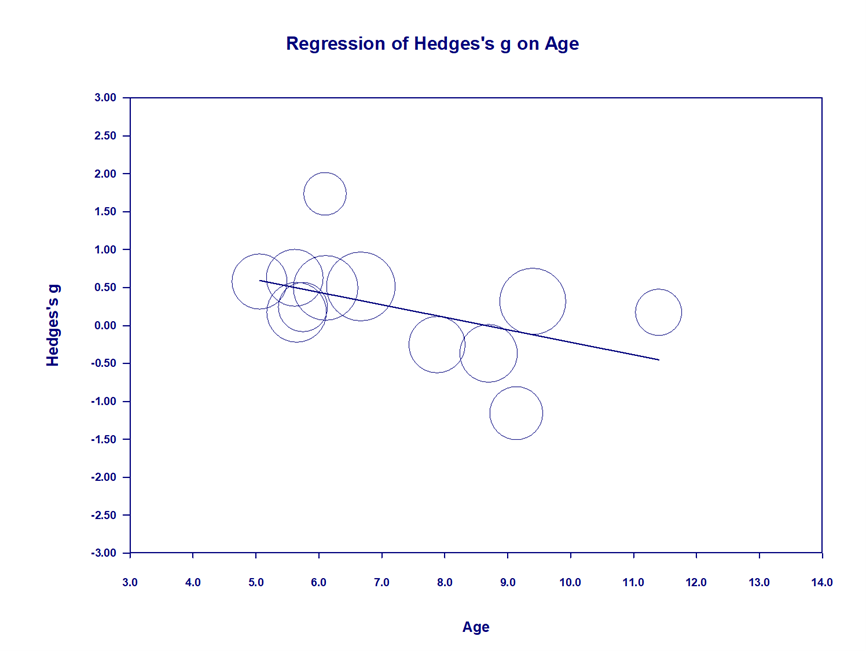

Supplement: S2 Fig — (TIF) [file pone.0269800.s002.tif]
