# Supplementary material for: The use of social robots with children and young people on the autism spectrum: A systematic review and meta-analysis
Source: PLoS One. 2022 Jun 22;17(6):e0269800. doi: 10.1371/journal.pone.0269800 (PMC9216612; doi:10.1371/journal.pone.0269800)
Supplement: S2 Table — (DOCX) [file pone.0269800.s004.docx]

**S2 Table. Quality assessment for included studies (maximum number of studies =40; RCT=17 and non-RCT =23)**

| **Quality**  **Rating** | **Selection**  **bias** | **Study design** | **Confounding**  **variable** | **Blinding** | **Data**  **collection** | **Withdrawal** |
| --- | --- | --- | --- | --- | --- | --- |
| Strong | 4 | 7 | 5 | 2 | 20 | 26 |
| Moderate | 30 | 31 | 6 | 31 | 16 | 2 |
| Weak | 6 | 2 | 29 | 7 | 4 | 12 |
| **RANDOMISED CONTROLLED TRIALS** | | | | | | |
| Strong | 2 | 6 | 2 | 1 | 13 | 12 |
| Moderate | 14 | 10 | 3 | 14 | 4 | 1 |
| Weak | 1 | 1 | 14 | 2 | 0 | 4 |
| **NON-RANDOMISED CONTROLLED TRIALS** | | | | | | |
| Strong | 2 | 1 | 3 | 1 | 7 | 14 |
| Moderate | 16 | 21 | 3 | 17 | 12 | 1 |
| Weak | 5 | 1 | 17 | 5 | 4 | 8 |
