# Supplementary material for: The use of social robots with children and young people on the autism spectrum: A systematic review and meta-analysis
Source: PLoS One. 2022 Jun 22;17(6):e0269800. doi: 10.1371/journal.pone.0269800 (PMC9216612; doi:10.1371/journal.pone.0269800)
Supplement: S3 Table — (DOCX) [file pone.0269800.s005.docx]

**S3 Table. Individual study quality assessment overview**

| **Reference** | **Global rating** | **Selection Bias** | **Study design** | **Confounders** | **Blinding** | **Data collection methods** | **Withdrawals/**  **Drop out** |
| --- | --- | --- | --- | --- | --- | --- | --- |
| **RANDOMISED CONTROLLED TRIALS** | | | | | | | |
| **Huskens et al., 2013**  **RCT** | Moderate | Moderate | Moderate | Weak | Moderate | Strong | Strong |
| **Marino et al., 2020**  **RCT** | Strong | Moderate | Strong | Weak | Moderate | Strong | Strong |
| **So et al., 2018a**  **RCT** | Weak | Moderate | Strong | Weak | Moderate | Moderate | Weak |
| **So et al., 2018b**  **RCT** | Weak | Moderate | Moderate | Weak | Moderate | Strong | Weak |
| **So et al., 2019a**  **RCT** | Weak | Weak | Moderate | Weak | Moderate | Strong | Weak |
| **So et al., 2019b**  **RCT** | Weak | Moderate | Moderate | Weak | Weak | Strong | Strong |
| **So et al., 2020a**  **RCT** | Weak | Moderate | Moderate | Weak | Weak | Strong | Strong |
| **So et al., 2020b** | Moderate | Moderate | Moderate | Moderate | Moderate | Strong | Strong |
| **Srinivasan et al., 2015a**  **Pilot RCT** | Moderate | Moderate | Strong | Weak | Moderate | Moderate | Weak |
| **Srinivasan et al., 2015b**  **Pilot RCT (overlapping sample)** | Moderate | Moderate | Strong | Weak | Moderate | Moderate | Weak |
| **Srinivasan et al., 2016a**  **Pilot RCT (overlapping sample)** | Moderate | Moderate | Strong | Weak | Moderate | Strong | Strong |
| **Srinivasan et al., 2016b**  **Pilot RCT (overlapping sample)** | Moderate | Moderate | Strong | Weak | Moderate | Strong | Strong |
| **Zheng et al., 2020**  **RCT** | Weak | Moderate | Moderate | Weak | Moderate | Strong | Weak |
| **De Korte et al., 2020**  **RCT** | Strong | Strong | Strong | Moderate | Strong | Strong | Strong |
| **Yun et al., 2017**  **RCT** | Strong | Moderate | Strong | Moderate | Moderate | Strong | Strong |
| **Costescu et al., 2017**  **RCT** | Moderate | Strong | Strong | Weak | Moderate | Moderate | Strong |
| **Pop et al., 2013a**  **RCT** | Strong | Moderate | Moderate | Strong | Moderate | Strong | Strong |
| **Pop et al., 2014**  **RCT** | Strong | Moderate | Moderate | Strong | Moderate | Strong | Strong |
| **Simut et al., 2016**  **RCT** | Moderate | Moderate | Moderate | Weak | Moderate | Moderate | Moderate |
| **Kim et al., 2013**  **RCT** | Moderate | Moderate | Moderate | Weak | Moderate | Strong | Strong |
| **Kim et al., 2015**  **RCT (overlapping sample)** | Moderate | Moderate | Moderate | Weak | Moderate | Strong | Strong |
| **NON-RANDOMISED CONTROLLED TRIALS** | | | | | | | |
| **Huskens et al., 2015** | Moderate | Weak | Moderate | Moderate | Moderate | Strong | Strong |
| **Kaboski et al., 2015** | Strong | Moderate | Moderate | Strong | Moderate | Strong | Strong |
| **So et al., 2016** | Weak | Weak | Moderate | Weak | Moderate | Weak | Weak |
| **Tapus et al., 2012** | Moderate | Moderate | Moderate | Weak | Moderate | Weak | Weak |
| **Warren et al., 2015** | Weak | Moderate | Moderate | Weak | Moderate | Moderate | Weak |
| **Zheng et al., 2016** | Weak | Moderate | Moderate | Weak | Weak | Moderate | Weak |
| **Kuzamaki et al., 2018a** | Moderate | Moderate | Moderate | Weak | Moderate | Moderate | Strong |
| **Kuzamaki et al., 2018b** | Moderate | Strong | Moderate | Weak | Moderate | Strong | Strong |
| **Yoshikawa et al., 2019** | Weak | Moderate | Moderate | Weak | Weak | Moderate | Strong |
| **Srinivasan et al., 2013** | Moderate | Moderate | Moderate | Weak | Moderate | Strong | Strong |
| **Srinivasan & Bhat, 2014** | Moderate | Moderate | Moderate | Weak | Moderate | Moderate | Strong |
| **Costa et al., 2018** | Moderate | Moderate | Moderate | Strong | Moderate | Moderate | Weak |
| **Duquette et al., 2008** | Weak | Weak | Moderate | Weak | Weak | Moderate | Strong |
| **Scassellati et al., 2018** | Weak | Moderate | Moderate | Weak | Weak | Moderate | Strong |
| **Pop et al., 2013b** | Strong | Moderate | Strong | Strong | Moderate | Strong | Strong |
| **Simut et al., 2012** | Moderate | Moderate | Moderate | Weak | Moderate | Moderate | Strong |
| **Vanderborght et al., 2012** | Moderate | Moderate | Moderate | Weak | Moderate | Moderate | Strong |
| **Silva et al., 2018** | Weak | Moderate | Moderate | Weak | Weak | Weak | Weak |
| **Silva et al., 2019** | Weak | Moderate | Moderate | Weak | Moderate | Strong | Weak |
| **Silva et al., 2020** | Moderate | Strong | Weak | Moderate | Strong | Strong | Moderate |
| **Puyon & Giannopulu, 2013** | Weak | Weak | Moderate | Weak | Moderate | Moderate | Strong |
| **Pierno et al., 2008** | Weak | Moderate | Moderate | Moderate | Moderate | Weak | Weak |
| **Giannopulu et al., 2014** | Weak | Weak | Moderate | Weak | Moderate | Moderate | Strong |
