# Supplementary material for: The use of social robots with children and young people on the autism spectrum: A systematic review and meta-analysis
Source: PLoS One. 2022 Jun 22;17(6):e0269800. doi: 10.1371/journal.pone.0269800 (PMC9216612; doi:10.1371/journal.pone.0269800)
Supplement: S4 Table — (DOCX) [file pone.0269800.s006.docx]

**S4 Table. Search terms per bibliographic database**

*Search strategy example: PubMed*

Autis* OR ASD OR ASC OR Asperger* OR “pervasive developmental disorder” OR PDD-NOS OR PDD-unspecified (tw)

**AND**

Robot* OR “human-robot” OR “human-machine” OR “robot-mediated” OR “robot-based” OR “robot-assisted” OR robot* n3 interv* OR robot* n3 therap* OR robot* n3 train* OR robot* n3 treatment OR robot* n3 management OR robot* n3 care OR robot* n3 support OR robot* n3 education (tw)

**AND**

Social OR emotion* OR communication OR educat* OR academ*OR behavio*r OR health (tw)
