# Supplementary material for: The use of social robots with children and young people on the autism spectrum: A systematic review and meta-analysis
Source: PLoS One. 2022 Jun 22;17(6):e0269800. doi: 10.1371/journal.pone.0269800 (PMC9216612; doi:10.1371/journal.pone.0269800)
Supplement: S5 Table — (DOCX) [file pone.0269800.s007.docx]

**S5 Table. Vote count outcomes by setting**

|  | **Positive outcome**  **(Skills development)** | | **No significant**  **difference** | | **Negative**  **Outcome** | |  |
| --- | --- | --- | --- | --- | --- | --- | --- |
| **Setting** | **RCT** | **Non-RCT** | **RCT** | **Non-RCT** | **RCT** | **Non-RCT** | **Total** |
| **Autism centre/clinic** | 4 | 6 | 3 | 2 | 0 | 0 | 15 |
| **Home** | 2 | 4 | 0 | 0 | 0 | 1 | 7 |
| **School** | 4 | 1 | 2 | 0 | 0 | 0 | 7 |
| **Laboratory** | 1 | 5 | 0 | 1 | 0 | 0 | 7 |
| **Robotics camp** | 0 | 0 | 0 | 1 | 0 | 0 | 1 |
| **Not reported** | 0 | 2 | 1 | 0 | 0 | 0 | 3 |
